# Uterine Primitive Neuroectodermal Tumor

**DOI:** 10.7759/cureus.16437

**Published:** 2021-07-17

**Authors:** Taysa Benitez Delgado, Maria Laseca-Modrego, Daniel Gonzalez Garcia-Cano, Andres Rave Ramirez, Octavio Arencibia-Sánchez

**Affiliations:** 1 Gynecologic Oncology, Complejo Hospitalario Universitario (C.H.U.) Insular-Materno Infantil, Las Palmas de Gran Canaria, ESP

**Keywords:** primitive, neuroectodermal, tumor, uterus, gynecology, ewing, sarcoma, oncology, prognosis

## Abstract

Uterine primitive neuroectodermal tumors (PNETs) are rare entities, with only around 70 cases published in the literature. Most of them are diagnosed in advanced stages with rapid progression and poor prognosis. Herein, we present a case of a 71-year-old patient with postmenopausal metrorrhagia and an ultrasound finding of endometrial thickening. The pathological diagnosis after an endometrial biopsy showed PNET. In the study of extension, possible distant dissemination with infiltration of the sigmoid and liver was observed. Chemotherapy treatment was proposed, but not begun due to the rapid progression of the disease. Four months after the initial diagnosis, the patient died of multiple organ failure. While there is no optimal chemotherapy treatment regimen for PNET, some studies have reported encouraging results. It is necessary to publish more studies emphasizing the follow-up and survival of the disease to establish which may be the best treatment option and thus improve the current poor prognosis.

## Introduction

Primitive neuroectodermal tumors (PNETs) are included in the family of Ewing's sarcomas [1]. They have a neuroectodermal origin as a consequence of the translocation (11.22) (q24; 12) that originates in the Ewing sarcoma breakpoint region 1 - friend leukemia integration 1 transcription factor (EWS-FLI1) gene [2]. Most are located in the central axis deriving from the neural tube in the brain or spinal cord, but the location in the uterine body is extremely rare [3]. Most of them are diagnosed in advanced stages and have a rapid progression with a poor prognosis. Standard treatment consists of surgery followed by radiation therapy and/or chemotherapy [4]. We present a clinical case of this rare entity.

## Case presentation

We present a 71-year-old woman with a personal history of rheumatoid arthritis, with no relevant family history. Regarding gynecological history, menarche at 12 years, G3C3, menopause at 50 years. She was referred to a gynecology clinic due to the accidental ultrasound finding of endometrial thickening during the study of recurrent urinary tract infections. The patient reported intermittent and scarce postmenopausal metrorrhagia for five months without any other associated symptoms. She was not using estrogen therapy. The patient did not perform routine gynecological examinations.

**Figure 1:**
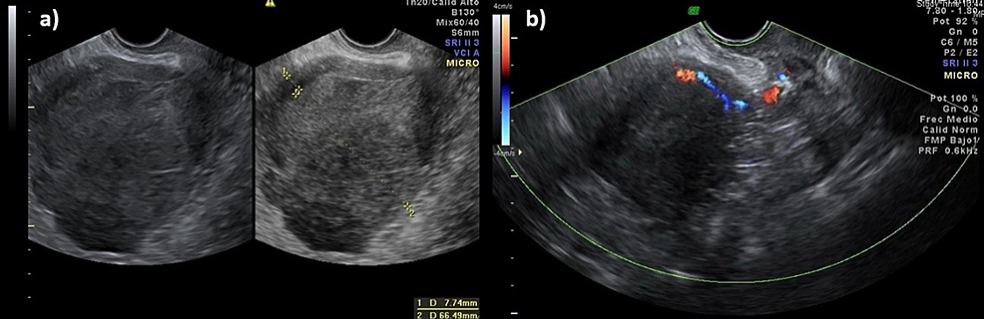
Transvaginal Doppler ultrasound A) Anteverted uterus enlarged with hysterometry of 107x73x90 mm, heterogeneous myometrial walls at the expense of subserous myoma at the level of the uterine fundus measuring 38x22x37 mm. 66mm thickened endometrium. A 3 mm thinning of the posterior myometrial face is observed with loss of the endometrial-myometrial interface. Endometrial/myometrial relationship 33/47 of the posterior face. The anterior myometrial thickness of 10 mm, showing the endometrial-myometrial interface. B) Endometrium with type two Doppler score on the anterior side.

On examination, she had a body mass index of 32. On gynecological examination, she had an enlarged uterus (12 cm). A Doppler ultrasound was performed, revealing an enlarged uterus with a 66 mm thickened endometrium, with a type two Doppler score on the anterior surface, compatible with the tumor. Also, a loss of the endometrial-myometrial interface was observed (Figure 1).

An endometrial biopsy was performed. The pathological diagnosis reported an undifferentiated malignant neoplasm compatible with PNET. The immunohistochemical study revealed positivity for the markers Vimentin and CD99.

**Figure 2:**
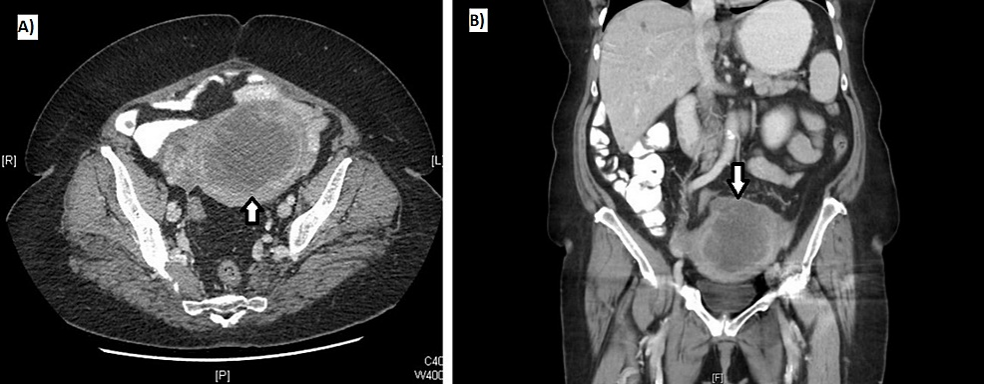
Abdominopelvic CT scan after administration of oral Gastografin and intravenous iodinated contrast in the venous phase A) and B) Anteverted uterus with great distention and occupation of the endometrial cavity by a mass with a neoplastic appearance that infiltrates more than 50% of the thickness of the myometrium, as well as the uterine serosa, with an exophytic growth pattern, which contacts a sigmoid segment, without fat cleavage plane and with small inflammatory changes in the adjacent fat, not being able to rule out tumor infiltration of the fat.

In the extension study, with computed tomography (CT) (Figure 2), findings compatible with endometrial neoplasia were observed, which appeared to surpass the serosa and contact the sigmoid, as well as possible liver metastases. Liver lesions were biopsied with a negative result.

An exploratory laparoscopy was indicated, which found the uterus totally infiltrated by neoplastic tissue that blocked the pelvis and appeared to infiltrate the sigmoid, liver lesions suggestive of metastasis, and a moderate amount of ascites.

Palliative treatment was proposed with chemotherapy based on vincristine, adriamycin, cyclophosphamide, ifosfamide, and etoposide (VAC/IE), but could not be initiated due to presenting symptoms of renal and respiratory failure with secondary sepsis progressing to multi-organ failure. The patient died four months after the diagnosis of the disease.

## Discussion

PNETs belong to a group of small round cell tumors that are most commonly found in the central nervous system, soft tissues, and bones. The origin in the female genital tract is rare. The most common location is the ovary, followed by the uterine body [3], vagina, and cervix [5].

To date, about 70 cases of uterine origin have been described in the entire literature [6,7]. The largest series of cases includes 17 [3]. Although these cases share the same diagnosis, they vary in their clinical presentation, treatment, and prognosis.

PNETs of the uterus have been described in all ages, although they are more frequent in adolescent or postmenopausal women [8]. Postmenopausal bleeding is usually the most common symptom, similar to endometrial adenocarcinoma. However, unlike endometrial cancer, which is usually diagnosed in the early stages, uterine PNET is more frequently diagnosed in advanced stages [1]. In the clinical case presented, the patient presented with postmenopausal bleeding, although she had not consulted previously for this reason; the diagnostic suspicion was a chance ultrasound finding. Despite the few symptoms, after completing the study, a PNET of uterine origin was diagnosed in an advanced stage.

The diagnosis of this entity requires histological confirmation and imaging tests or surgery to complete the staging. In the series of 17 cases [3], 12 of the included patients underwent surgery or imaging to determine the stage, although only five underwent biopsy. In our case, for the histological study, a single biopsy was taken since she was not a candidate for primary surgery.

The differential diagnosis of uterine PNETs includes tumors that present neuroectodermal elements found in the central nervous system. Diagnosis is based on microscopic and immunohistochemical evidence of neuroectodermal differentiation with markers such as CD99, friend leukemia integration 1 transcription factor (FLI1), and vimentin [9]. The most specific marker for PNET is CD99 [10]. In our case, both markers, the CD99 marker, and Vimentin marker were positive. In the case series [3] of nine cases in which the CD99 marker was performed, seven were positive. PNETs originate from the t (11; 22) q24q12 translocation in the Ewing sarcoma gene, which 90% of the time originate from the Ewing sarcoma breakpoint region 1 - RNA binding protein 1 (EWSR1)/FLI1 fusion gene [2]. However, few cases of uterine PNETs have been described that present this gene in the literature [3,11]. In the case presented, it was raised, but the fluorescent in situ hybridization (FISH) study could not be carried out for its determination.

Given the scarcity of published cases, there is no consensus to establish an optimal treatment regimen in uterine PNET. As a standard, the described treatment has been surgery followed by chemotherapy and/or radiotherapy. Patients treated with surgery and radiotherapy have a relapse rate close to 90% [11]. Multimodal therapy improves disease-free survival, but the optimal chemotherapy treatment regimen has not been described.

Akazawa et al. conducted a search in the medical literature analysis and retrieval system online (MEDLINE) database, obtaining 178 papers, of which finally 49 cases of uterine PNETs were included. They analyzed the chemotherapy regimen that each patient received, concluding that the determining factor for choosing one treatment over another is age. They found that the Ewing sarcoma regimen [vincristine, doxorubicin, cyclophosphamide/ ifosfamide, etoposide (VDCIE), or vincristine, doxorubicin, cyclophosphamide, actinomycin-D (VDCA)] was used more frequently in young patients. However, less than half of the patients older than 60 years received any of these mentioned drugs. The frequent use of platinum derivatives did not change according to age groups which are used in half of them along with other chemotherapeutic agents. The initial treatment plan was palliative chemotherapy based on VAC/IE in the case presented. However, it could not be initiated and proven effective due to the rapid progression of the disease.

Most of the published works focus on the diagnosis and choice of initial treatment, without referring to the prognosis and survival of the disease. Younger patients have a better prognosis with a 75% survival at two years, compared to 32% in the group of postmenopausal women [6]. In one of the most recently published cases [12], the patient had a very good response to systemic treatment with chemotherapy after disease progression with multiple distant metastases. Despite residual disease on imaging, she was symptom-free for two years after the initial diagnosis.

## Conclusions

Although cases have been published where the evolution has been more favorable, the majority, as with the patient in this study, have presented a poor prognosis. There are no data on whether routine gynecological examinations can improve the detection or prognosis of these types of rare diseases. Likewise, the genetic study, although it can provide valuable information, in tumors of this type in the uterus is not very clarifying since, according to the series of cases studied, the presence of recognizable mutations is rare. 

In order to offer new treatment alternatives, it is necessary to publish all the cases in which this entity is diagnosed, with greater emphasis on follow-up, as well as response to the therapy received. Only that way we will be able to advance in the knowledge of this rare entity.
